# Health systems resilience and private for-profit sector engagement: lessons from the second COVID-19 wave in Uttar Pradesh, India

**DOI:** 10.1093/heapol/czag001

**Published:** 2026-01-13

**Authors:** Ankita Meghani, Shreya Hariyani, Prabhjeet Singh, Sara Bennett

**Affiliations:** Department of International Health, Johns Hopkins Bloomberg School of Public Health, 615 N. Wolfe St, Baltimore, MD 21205, United States; Johns Hopkins India Private Limited (JHIPL), G Block, C1 Shopping Arcade, Konark Estate, Connaught Rd, Pune 411001, India; Department of International Health, Johns Hopkins Bloomberg School of Public Health, 615 N. Wolfe St, Baltimore, MD 21205, United States; Department of International Health, Johns Hopkins Bloomberg School of Public Health, 615 N. Wolfe St, Baltimore, MD 21205, United States

**Keywords:** health systems research, informal sector, markets, private providers, qualitative research, equity

## Abstract

India's second wave of the COVID-19 pandemic in April–June 2021 involved an explosion of case numbers, with devastating consequences for the country’s already strained health systems. This case study examines the private health market response to the pandemic in Uttar Pradesh, India’s most populous state. We analyzed 203 news articles to understand both the experiences of private providers and patients in response to government policies being implemented in the state. This analysis informed our interviews with 20 state-level officials, district-level key informants, and formal and informal private for-profit providers across three districts. We found that private-sector hospitals were rapidly engaged to manage a surge in new infections and severe cases, but private bed capacity quickly filled, causing patients to be turned away. Informal private providers played a vital role in rural areas, serving as round-the-clock care sources. However, the news media reported inadequate medical care from such providers leading to COVID-19-related deaths. Access to reliable information on COVID-19 was challenging and social media became a platform for citizens and providers to share information about available resources, treatment, and COVID-19 management. However, misinformation also spread. While the government attempted to counter misinformation and regulate private hospitals, challenges persisted in providing and accessing accurate information. Oxygen and drug supply challenges also emerged, with private hospitals requiring patients to arrange for oxygen themselves due to scarcity. To address this and rising costs of care, the government issued price caps, monitored overcharging, and regulated drug and oxygen distribution. Government schemes also attempted to provide insurance for both public and private health workers, however, awareness and implementation of such schemes were inadequate. Policymakers should develop mechanisms to engage, or where relevant, integrate all private for-profit providers onto a common platform, strengthen referral linkages amongst them, and support communities of practice to increase awareness of government health policies and improve the implementation of government schemes. Such measures would help facilitate equitable access to care and help manage current health needs and future health emergencies.

Key messages

- Private health markets continued to play an important role in serving COVID-19 patients during the second wave of the pandemic in Uttar Pradesh. Specifically, engagement of the formal private health sector increased as district health authorities enlisted private hospitals to boost COVID-19 surge capacity.
- Adaptations and learning from private testing centers helped strengthen testing and reporting during the Delta wave. However, more planning, earlier engagement of the private sector, and greater outreach to rural areas with limited public infrastructure would have improved the response.
- Collaboration between public and private for-profit sectors resulted in improved data sharing on COVID-19 cases and outcomes, and aided resource allocation for oxygen and beds. However, equity concerns emerged regarding access to COVID-19 services among rural populations due to the exclusion of informal private providers.
- Reports of private hospitals passing on increased costs to patients despite price caps highlighted regulatory gaps, however, there are examples of government holding those in violation accountable.
- Efforts to integrate all private providers, bridge government–provider information gaps, and strengthen communities of practice are needed for more effective and equitable access to care.

## Introduction

The COVID-19 pandemic challenged health system resilience across the globe, with many health systems struggling to respond to the pandemic while maintaining essential health services. Between April to June 2021, the second wave of the pandemic (Delta strain) rapidly spread across India putting health services across the country under severe strain. News reports described shortages in medical oxygen, hospital beds, and COVID-19 therapeutics, and an overworked health and medical workforce (Ratna et al. 2021). As health systems became overwhelmed, people turned to online platforms such as Twitter (now X) to crowdsource information about available hospital beds and oxygen. India reached historically high numbers of daily cases of ∼400 000 cases per day (7-days moving average) in the first week of May 2021 (DePasquale et al. 2021). A United Nations report estimated that 240 000 lives were lost in India between April and June 2021 due to the Delta variant (United Nations 2022), however this number is likely underreported (Menon et al. 2019). A recent analysis estimated that India’s COVID-19 death toll was likely underreported 8–10-fold compared with the official counts, corresponding to 2.8–5.2 million excess deaths during April 2020–June 2021 (Banaji and Gupta 2022).

In prior work (Meghani et al. 2022), we documented the way in which the Government of Uttar Pradesh (UP) sought to coordinate and collaborate with the private for-profit health sector during the first wave of the COVID-19 pandemic. In this paper, we broaden our analysis to consider not only issues around public and private sector coordination, but also the overall functioning of the private for-profit health market in UP, India and how this affected health system resilience.

Kruk et al. (2015, 2017) suggest that the characteristics of a resilient health system are that it is integrated, adaptive, aware, self-regulating, and diverse (Kruk et al. 2015, 2017). We focus on two of these aspects of resilience (integrated and adaptive) that are particularly relevant to health markets and to our case study. The concept of integration refers to the ability of a health system to work across sectors and coordinate between government and private-sector actors. Adaptive characteristics include the capacity to act on evidence and feedback so as to improve performance over time. The Delta wave in India occurred at a point when decision-makers in stakeholders in India had already experienced the first wave of the pandemic, and could potentially learn from other countries with regard to the Delta wave.

**Figure 1:**
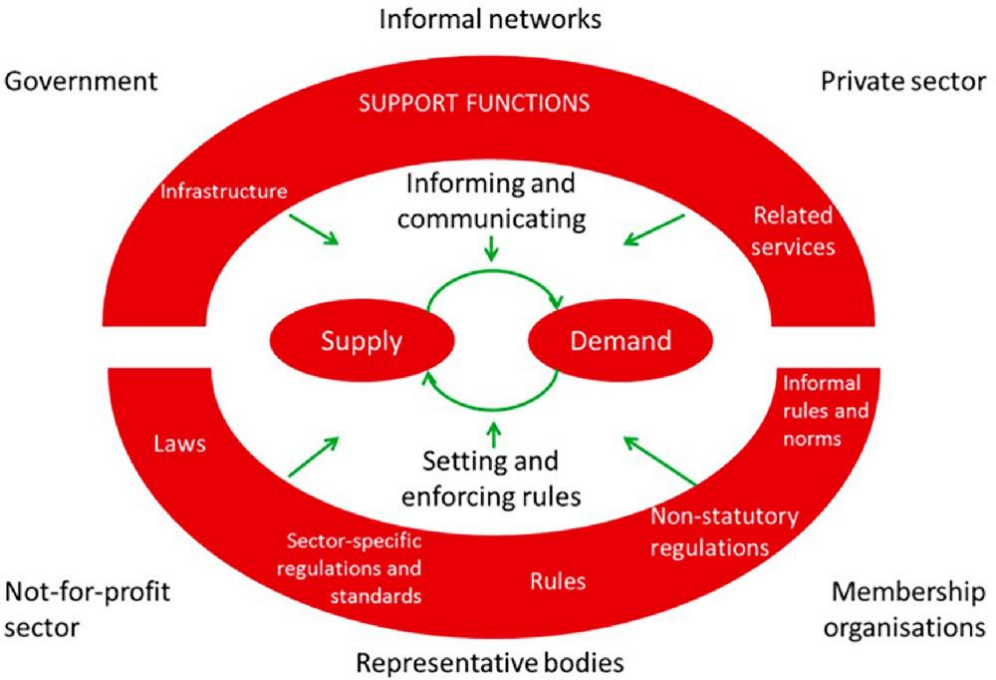
Conceptual framework for pluralistic health markets.

In pursuing this analysis, we drew upon a conceptual framework developed by Bloom et al. (2014a, 2014b) to analyze pluralistic health markets (Fig. 1) (Bloom et al. 2014b). As Fig. 1 outlines, at the core of the market is the exchange between supply (both government and private health care providers) and demand (clients). The relationship between supply and demand is mediated by information and communication (e.g. what clients learn about the relative expertise of providers, or their ability to access supplies), as well as by the market rules established by government and professional organizations. The upper shaded outer ring in Fig. 1 depicts support functions such as the procurement of essential supplies and inputs (oxygen, ventilators, testing) as well as infrastructure, administrative, and information systems. The lower shaded area outlines the variety of rules and norms, both formal and informal, that govern how the market works. Finally, stakeholders in the market, from government to private providers, networks, and associations, are depicted around the outside. All of these factors influence the performance of health markets, reflected by the number, type, quality, costs, and effectiveness of health services provided and received.

UP’s health market is highly pluralistic and relies on a diverse array of non-government actors. The private health sector remains the dominant source of care for all Indians, including the urban and rural poor in UP—48% Indians in the lowest health quintile use the private sector as the first point of care (The DHS Program 2016). The UP private for-profit health market consists of a broad set of providers ranging from rural medical practitioners (RMPs) or informal providers, who are experience-based practitioners with no formal medical or health training, to alternative medicine practitioners of ayurveda, yoga, unani, siddha and homeopathy (AYUSH), and doctors with training in allopathic medicine (typically Bachelor of Medicine and Bachelor of Surgery, MBBS). These private for-profit providers, particularly informal and alternative providers, and allopathic private providers with solo clinics, were an important source of care during the first wave of COVID-19 (Gummidi et al. 2020, Rao et al. 2021, Meghani et al. 2022). Critical challenges, such as non-transparent pricing (Bloom et al. 2014a), overregulation of the private sector (Mouraviev and Kakabadse 2015), and a complete disregard of RMPs (Meghani et al. 2022), however, shaped how and who could access quality care for COVID-19 in UP’s health markets.

Our study seeks to analyze how different aspects of the private for-profit health care market in UP responded to the Delta wave of COVID-19. Within this broad objective we seek to understand where an integrated response supported overall health systems resilience and where it failed. Further, we reflect on the adaptive nature of the health system and where learnings from the first wave and elsewhere informed the appropriateness of the response. We hope that findings from this study will help governments, private providers, and civil society better understand how to make health services more affordable and accessible, particularly for the poor.

## Methods

To conduct this case study, we employed two data sources: (i) news media articles, and (ii) in-depth interviews with state and district level key informants and private providers in three districts of eastern UP. News media analysis was conducted to better understand the broader health context in UP and inform the development of the in-depth interview guides. Given the difficulties of interviewing people who sought care during the peak of the pandemic, we used news media analysis, in part, as a means to source local voices, including the perspectives of those seeking care.

### News-media analysis

We analyzed Hindi and English news articles, including blogs and letters to editors, from 1 January–1 July 2021 to: (i) describe patients’ experiences accessing COVID-19 services in the private for-profit health sector; (ii) document new and revised policies in the government’s approach to engaging the private sector during the second wave; and (iii) identify and describe the types of profiteering and other challenges in the private for-profit health sector during the COVID-19 pandemic.

News sources in three databases Access World News Research Collection, Nexis Uni, and ProQuest International Newsstream were searched for three terms ‘COVID-19,’ ‘Uttar Pradesh,’ and ‘private sector.’ The same searches were run in the GoogleNews database for Hindi articles sourced from local periodicals. Articles were included if they described the role of the private for-profit health sector and were excluded if they were not about health providers in the private for-profit sector or described experiences in states other than UP. We found 203 Hindi and English news stories eligible for analysis. Information was extracted on: (i) article details (e.g. type of news article, title, author’s name), (ii) the nature of the private sector, (iii) policy guidelines and initiatives involving the private sector, and (iv) patients’ experiences accessing care in the private sector. The specific items that were extracted for each category are available in the online supplementary material. When questions arose relating to categorization of the extracted data, the team collectively discussed them during weekly team calls to ensure consistent interpretation. In advance of coding all the news media articles, the team coded a subset of articles, discussed any discrepancies, and revised the categories of coding framework as needed.

### In-depth interviews

Building upon our first round of data collection in 2020 (Meghani et al 2022), we conducted a second round of interviews in Prayagraj and Gorakhpur. These two districts were originally selected because they were largely rural districts with a high caseload of COVID-19 that during the pandemic experienced a large influx of migrants returning home. We also conducted interviews in Deoria, a district neighboring Gorakhpur, in order to capture a larger sample of RMPs whom we had trouble identifying in our focal districts since RMPs are not officially recognized as providers in India. However, we located a cluster of practitioners near the Gorakhpur–Deoria border, and this area, like Gorakhpur, is largely rural, distant from the state capital, and borders Bihar.

A total of 20 interviews were conducted in this round including 4 key informant interviews at the state level and 1 at the district level (Table 1). The key informant interview guide focused on understanding: (i) changes to the government’s approach in engaging the private for-profit sector; (ii) how the government addressed shortages in hospital beds, oxygen, and COVID-19 therapeutics, as well as human resources; and (iii) new policies or guidelines targeting the private for-profit sector’s role during the second wave of the pandemic. In all, 15 private for-profit provider interviews were conducted across these three districts with allopathic providers, AYUSH providers, laboratory-service providers, and RMPs. The in-depth interviews sought to understand how these providers had adapted their practices during the Delta wave; sources of information they had relied on; their experiences in accessing supplies and technical information, and data reporting; and the nature of support they received from government. Strategies used to identify potential respondents have been described separately (Meghani et al. 2022).

**Table 1:** Study respondents at the district and state level.

Category of respondent	State-level	District-level
Government	1	1
Technical partners	1	—
Allopathic providers	—	6
AYUSH providers, including association representatives	1	3
Rural medical practitioners		4
Laboratory services (managers and pathologists)	1	2
Total	**4**	**16**

^a^Respondent was a representative of the AYUSH association.

^b^One respondent managed laboratory services, and another was a pathologist running a private laboratory.

Interviews were conducted in Hindi and English by phone or WhatsApp and in-person from September 2021–January 2022, ranging from 25 min to 1.5 h. Written informed consent was requested prior to each interview. Recordings were transcribed and translated into English, and following each interview, the team debriefed to discuss emerging themes and follow-up questions.

Transcripts from the interviews were analyzed thematically using framework analysis (Gale et al. 2013). The analytical framework followed five categories: (i) government’s approach to engaging private for-profit health providers in the second wave; (ii) providers’ experiences during the second wave, including barriers and facilitators; (iii) key sources of information about the pandemic; and (iv) patients’ experiences with accessing care and (v) profiteering and challenges associated with it. We applied this analytical framework to all interview data for private providers and state-level respondents and wrote memos for each category. These memos incorporated data from the news media analysis where relevant and drew on memos from the first round of interviews to document the differences in respondents’ experiences between the first and second wave to identify any learnings or adaptations that may have taken place.

### Ethical considerations

The authors’ institute deemed this study as non-human subjects research. Ethical approval for the research was obtained from the Institutional Review Board of SIGMA Research and Consulting, New Delhi, India (10016/IRB/20-21). Informed written consent was obtained from all participants prior to interview, including consent for audio recording. If participants did not provide permission to audio record, detailed handwritten notes were taken instead.

## Results

Results are organized according to the different aspects of the pluralistic health market framework shown in Fig. 1, focusing on the private for-profit health sector (hereafter referred to as the ‘private sector’). This includes service supply and demand; information and communication; support services; and norms and regulations, such as service standards. In subsequent sections, we assess the nature of (i) integration and (ii) adaptation in the UP health system, and the extent to which these factors supported the pandemic response.

### Supply and demand for services

#### COVID-19 treatment and care

During the first wave of the pandemic, public-sector facilities were primarily responsible for providing COVID-19 treatment while private-sector hospitals were viewed as a ‘back-up’ and prepared as surge capacity. Only private-sector facilities approved by government as dedicated COVID-19 facilities could provide COVID-19 treatment services. During the Delta wave, the sharp rise in severe cases meant that public-sector capacity was quickly exhausted and public hospitals began reporting non-availability of beds in early April 2021, as well as a shortage of medical staff (Sharma 2021). As a result, the government began rapidly designating private hospitals, typically located in city centers, as COVID-19 facilities.

State-level key informants acknowledged that the nature of the second wave made private providers more central to the response, as the government was essentially ‘firefighting.’

‘During the second wave instead of the response being completely managed by the public health care side, there was a deeper involvement of private sector.’ (State-level key informant 01)

However, private-sector capacity was also quickly overwhelmed, resulting in patients being turned away. News sources reported numerous incidents of patients going from hospital to hospital seeking oxygen-equipped beds in intensive care units, and, in many cases, losing their lives in the process (TNN 2021).

In rural areas, RMPs remained the first point of care during the second wave. Patients reportedly had no option but to go to RMPs because of closed government facilities and their inability to travel to the city to access care from more expensive formal private providers. Despite limited training, they offered round-the-clock support to patients from the community and neighboring villages throughout the second wave, and frequently were the only source of care locally.

‘If we talk about the Corona period, we had 24 h duty because all the popular and well to do doctors had backed off. They all closed down.’- (RMP 03, Gorakhpur)

A news article similarly highlighted the perspective of a farmworker in a district in UP:

‘The village quacks invoke more confidence among the people than our healthcare system,’ said Pandey, 49, an activist for farmworkers. ‘They treat patients sincerely and attend to them when people need reassurance. People believe that once you are admitted to a government hospital, you die.’ (Pierson and Parth 2021)

When many pharmacies in rural areas shut during lockdowns, RMPs also served as medical dispensaries, providing patients with glucose, paracetamol, and other medicines to treat flu-like symptoms. RMPs recommended that patients go to the nearest government facility to seek care if symptoms were severe or did not resolve; however, many patients who sought care from RMPs reportedly succumbed to COVID-19 due to lack of access to oxygen and inadequate medical care (Rashid 2021a, The Daily Telegraph 2021).

Several news articles (Hindustan Times 2021, Rashid 2021a, Yashee 2021) described how rural patients avoided seeking care in government facilities because they feared a positive test result would mean being removed from their homes and families. RMPs did not require their patients to get tested for COVID-19 when seeking care from them, perhaps due to a lack of awareness resulting from their exclusion from the formal COVID-19 health response.

#### COVID-19 testing

Overall, respondents from both government and private sector diagnostic firms indicated that testing was timely and capacity was sufficient, largely because testing was significantly ramped up during the first wave, which aided preparedness during the second wave. However, media analysis from the peak of the second round indicates that testing capacity struggled to keep up, e.g. test results often took 2 days to come back (Bhaskar 2021). This was confirmed by a provider who noted that given the delay in receiving tests, patients often left or went to another facility, making it difficult to communicate test results to them (District-level formal private provider 08, Gorakhpur).

By several accounts people living in rural areas were significantly disadvantaged. For example, PCR tests were difficult to come by in rural areas and hence people tended to rely instead on rapid tests. One private diagnostics company noted that though it tried to provide services outside of cities, its ability to do so was hampered by poor transport networks (Private provider 02, Prayagraj). There were widespread media reports of challenges with getting tested in rural UP due to public health facilities being closed, long distances to testing centers, and lack of public health outreach to conduct testing (Hindustan Times 2021). Another news article suggested that RMPs further undermined testing by indicating to clients that there was no need to get tested (The Daily Telegraph 2021).

### Support functions

Multiple failures materialized in the market around support functions. For example, private diagnostic providers noted challenges relating to shortages of personal protective equipment and medium to store samples as well as difficulties in transporting samples (Private provider 02, Prayagraj). We focus on two domains, oxygen and drug supply, which became central constraints in accessing care given the nature of the Delta wave.

#### Oxygen

Private providers indicated that the main constraint was not bed capacity, but rather oxygen shortages, as reported in multiple news articles (Rashid 2021b, Wallen 2021).

‘While the government had data and ICU [intensive care unit] beds were available in private hospitals, the key challenge was the lack of oxygen, so beds without oxygen in the private sector were also of no use.’ (Private provider 03, Gorakhpur)

In urban and peri-urban areas, news sources reported daily on facilities running out of oxygen supply (Parkin 2021). Private hospitals had beds available but asked patients’ families to arrange for their own oxygen, many of them resorting to the black market (Krishnan 2021, Wallen 2021). Numerous accounts emerged of patients being strapped to oxygen cylinders while looking for a hospital that would admit them; many patients reportedly died while seeking care (Covid in Uttar Pradesh 2021). Private providers in study districts reported:

‘If patients were able to source their own oxygen, then they would admit them. People used to carry the oxygen on a bike. People were trying their best to get the oxygen. Cost was not a factor. People used to pay whatever they have. People used to buy it for any price as it was hardly available. Oxygen was Rs. 700 to Rs. 7000.’ (Private provider 13, Gorakhpur)

At the height of the second wave, private providers also noted the initial difficulty in procuring oxygen concentrators, oxygen masks, and oxygen cylinders for their facilities, both in terms of availability and cost.

‘Oxygen concentrators were not available. We had paid 100% upfront and then it would come in 10–15 days and suddenly they said that the brand is not available now, we need to buy another brand’s concentrator, which would cost twice the previous one. Everything got streamlined in 10–15 days, but for 10–15 days there were problems.’ (Private provider 02, Gorakhpur)

To address challenges with oxygen shortages, at the national level, the Government of India under oversight of the supreme court worked to ramp up the central pool of oxygen (Rautray 2021). Oxygen availability was assessed hourly in order to allocate oxygen to states as per their needs. State governments were expected to pick up and distribute supplies based on need to public and private facilities. Ensuring a rational allocation of medical oxygen however was not straightforward, partly because of the plethora of players in the market.

‘Government did try to manage that but the whole eco system was filled with different types of players both in organized and unorganized area so it took some time for the government to understand and rationally distribute this oxygen’ (District level key informant 01, Gorakhpur)

The UP state government ended up rationing oxygen based on the number of cases in different facilities both public and private. Typically, larger facilities, such as tertiary hospitals and dedicated Covid hospitals received more oxygen because they served more severe COVID-19 patients who required oxygen-supported beds.

The ultimate responsibility for monitoring and distributing oxygen lay with the district-level administration. The Government created a portal to receive oxygen requirement data from facilities. Nodal doctors were appointed in each facility to ascertain the number of patients being admitted for COVID-19 and the level of care required. They were also responsible for monitoring oxygen amounts being issued, used, and remaining in the hospital.

‘Every day they [the government] had put up in the portal, they [the government] put up an excel sheet which we [nodal doctors] had to fill regarding the number of patients we had on ventilators, the number of patients who are on bipap [a type of non-invasive ventilation] and the number of patients who were on high-flow nasal cannula and the number of patients who were only on oxygen…’ (Private provider 08, Gorakhpur)

Overall, private providers felt that the district-level administration was effective at managing the situation after the initial challenges.

‘Because the oxygen was directly monitored by the local administration, they made sure there was no shortage for surgical cases and for emergency cases.’ (Private provider 02, Gorakhpur)

## Drugs and commodities

Like oxygen, the unprecedented surge and severity of Delta strain cases put pressure on the availability of drugs and commodities.

‘There was a chaos and confusion leading to hoarding of drugs causing drug stock outs including essential basic drugs like paracetamol’ (Private provider 01, Prayagraj)

The national government provided emergency use authorization for the broad spectrum antiviral remdesivir, advising that it only be used in select moderate or severe hospitalized COVID-19 patients on supplemental oxygen. With limited availability of remdesivir, private hospitals left the task of securing the prescribed drug to the patients’ families. News media sources reported low availability, price hikes, and black-marketing of the drug (Wallen 2021). As a private hospital manager in one study district described:

‘Like we used to make a demand for remdesivir and give it to the attendants, those people bought it from outside at twice or triple the rate’ (Private provider 01, Prayagraj)

The government once again stepped in to regulate the situation after the initial crisis. Remdesivir was only to be purchased from licensed manufacturers and its distribution and use was heavily monitored by the government (PIB Delhi 2021). The UP state government also announced free remdesivir for patients admitted to public and private hospitals, with the district administration being responsible for distribution (IANS 2021).

### Information and communication

Access to reliable COVID-19 information posed a serious challenge. Although the government launched COVID-19 helplines, online portals listing public and private facilities, and posted signage on bed availability, patients continued to go from hospital to hospital in search of care due to unreliable phone lines and frequently outdated or inaccurate facility information (Lucknow Bureau, 2021; Pandey, 2021). Consequently, social media became a key platform where citizens exchanged information about the availability of care and commodities, as well as self-treatment and management information. However, such sources were also not always reliable and sometimes provided misinformation. For example, Ayurvedic gurus, such as Baba Ramdev, falsely advertised the emergency use of ‘coronil’ to treat COVID-19 (Godbole 2021).

Sourcing care information from social media, many patients who had managed to secure oxygen concentrators opted to use them at home; they only sought care when their oxygen saturation levels were dangerously low (Wallen 2021). A private provider, attributed the main cause of COVID-19-related mortality to delayed care-seeking, stating that ‘maximum mortality took place not because people reported to the hospital, it happened because people did not report to the hospital’ (District-level private provider 04, Prayagraj).

While systematic strategies to counter misinformation and guidance on managing COVID-19 at home were lacking, there were reports of government taking action against private hospitals. Private hospitals were accused of spreading ‘rumours’ about patients running out of oxygen and medicines (Crawford 2021) and in one extreme case, a district magistrate reportedly issued an order to take the strictest action against such hospitals (Hindustan Times 2021, Rashid 2021a, Yashee 2021). However, such government actions may have been based on spurious grounds given how oxygen and medical shortages hindered private hospitals from delivering adequate care (Haider 2021).

### Norms and regulation

Prior to the Delta wave, government had recognized multiple forms of market failure including: lack of access to technical guidance; price hikes by private providers; unaffordability of services; and absenteeism by health workers, and had sought to address these failures through technical guidance, new regulations, and government schemes (Meghani et al. 2022). This section focuses on four critical challenges and examines how they were addressed during the Delta wave.

#### Technical guidance and service standards

As during the first wave, allopathic and AYUSH providers relied on guidance released by the government, technical bodies like the Indian Council of Medical Research, and professional health associations such as the Indian Medical Association (IMA) and the National Integrated Medical Association. Government circulars and additional state- and district-level guidelines or memos were forwarded through WhatsApp. Webinars from district- and national-level health authorities, professional bodies, and technical agencies were conducted routinely to share updates and changes in COVID-19 treatment protocols. Particularly during strict lockdowns in the second wave, relying on web-platforms allowed doctors to connect with experts who specialized in different fields to understand how to manage COVID-19 in patients who had special needs, e.g. pregnant women, diabetic patients, and those presenting with high blood pressure.

Allopathic providers underscored the importance of peer-to-peer learning, drawing on their professional networks within the districts and colleagues who had initial experiences with the second wave to learn about treatment practices and outcomes. Unlike their allopathic counterparts, AYUSH providers felt that peer-to-peer learning opportunities were limited, however they continued to receive information through webinars, Whatsapp forwards, as well as print and televised news.

For RMPs, social media and news were the primary sources of technical information during the second wave. They received no formal communication from the government, nor were their professional bodies, such as the Indian Rural Medicos Society, disseminating information. District and state health authorities made no contact. In the absence of any formal dissemination of information, RMPs drew upon their informal networks with allopathic and AYUSH providers, whom they had previously worked with as paramedical staff, for medical advice. RMPs were generally familiar with safety protocols including maintaining distance, wearing masks, and using sanitizers.

#### Treatment charges and price caps

Government started imposing price caps on private-sector services during the first wave of the pandemic, but despite such price caps being in force, exorbitant and arbitrary charges for treatment in private hospitals emerged as a key challenge during the second wave. Numerous personal accounts of patients’ families being handed bills with unexplained and inflated charges were reported (Chaudhary 2021, Iftikhar and Sarfaraz 2021). Private providers also noted similar practices.

‘I was not a part of the Covid hospital. But a lot of hospitals who even didn't have much area have taken permission for it, and they were doing a lot of hanky-panky things to extort money from people. So, a lot of people in Covid, had also made a lot of money’ (Private provider 02, Gorakhpur)

To manage this, the district governments re-issued strict orders for adherence to stated price caps. Hospital bills were to be drawn in triplicate with one copy sent to the District’s chief medical officer to monitor. The bill had to include justification for charges and if the facility charged more than the prescribed rates, legal action would be taken. District administration received many complaints from citizens over hospital charges and several articles reported action being taken against facilities by the district administration (Chauhan 2021, Express News Service 2021, PTI 2021). Providers confirmed that district authorities were closely monitoring the situation.

‘There were CCTV recordings controlled by the District Magistrate to act as a check for private providers.’ (Private provider 01, Deoria)

In one district, the IMA worked closely with the administration to address the issue.

‘Overcharging happened and we have received complaints and I have also seen it… so wherever we felt there was a mess we considered it and ensured that the money was refunded on the direction of the government as well.’ (Private provider 05, Gorakhpur)

While providers viewed the price caps as fair from the patients’ perspective, they highlighted financial challenges in running facilities. For example, they noted that nursing and paramedical staff were being paid three times their normal salaries and many of the facilities had not fully recovered from the losses during the first wave. State-level key informants also acknowledged the challenges in running private facilities and were aware that private hospitals were likely facing much higher costs, especially given the shortage of health workers willing to work.

#### Empanelment of private hospitals

Several private hospitals that were empaneled for COVID-19 services were not under Ayushman Bharat (the government health insurance scheme for poor households), leaving vulnerable patients in a difficult situation. Providers attributed this to the burdensome processes entailed in becoming an Ayushman Bharat-empaneled hospital and low reimbursement rates under the scheme (Private provider 05, Gorakhpur).

‘I think it is the rate switch which is hindering because if the rates will not be proper the hospitals are not interested in those patients. If the private hospitals invest a lot of resources in setting up a healthcare unit and if it does get patients and the government is not ready to allow you to charge more or give you that much that the other insurance companies are paying us, then it doesn’t make any sense to have that [Ayushman Bharat] scheme.’ (Private provider 08, Gorakhpur)

To meet demands, district officials often proactively supported Ayushman Bharat-emapaneled private hospitals to become eligible to provide COVID-19 services. These private hospitals could receive approval within 24 h if their inspected facilities met the government requirements for delivering COVID-19 services.

#### Pradhan Mantri Garib Kalyan package and other incentives for health workers

On 24 April 2021, the government revived the Pradhan Mantri Gareeb Kalyan Yojana package, an accident insurance scheme that covered both government and health workers in the private sector for death due to COVID-19 and accidental death while on COVID-19 duty (National Portal of India 2021). Though government-announced, few allopathic providers were aware of the insurance policy, and many smaller hospitals, nursing homes, and laboratories established their own schemes should their workers get ill or die from COVID-19.

It was often difficult for affected families to claim from the scheme. An allopathic doctor who ran a COVID hospital in one of the study districts recounted the experience of a doctor’s family following his COVID-19-related death:

‘The government we approached them for some financial help because he was the only earning member of his family and in his early fifties but there was no response and in fact that letter we received they flatly refused that we are not giving anything to the private doctors.’ (Private provider 08, Gorakhpur)

This frustration was also noted by government staff—especially families of contractual workers who were seeking to access financial support following a COVID-19-related death. Challenges in making claims came from difficult-to-meet terms and conditions—such as having a positive test report for those who had died from COVID-19. To support frontline workers directly involved in the COVID-19 response, on 6 May 2021, the UP cabinet approved a 25% incentive to doctors, paramedics, other support staff—both from the private and public sector—on top of the salary or honorarium they were being paid (Janam Web Desk 2021). However, according to our respondents this policy was not being implemented in public or private sectors.

### Adaptation and learning

There were clearly important positive adaptations ahead of the second wave of the pandemic that reflected learnings by government. For example, during the first wave, private testing centers were frustrated with the government databases used to upload test results. However, the processes were streamlined before the Delta wave. Overall health staff were better prepared and well versed in how to respond. Response to the Delta wave was more decentralized compared to the first wave, with district administration playing a key role in engaging and regulating the private sector, and this enabled greater responsiveness to local conditions. Furthermore, testing processes and capacity were strengthened during the first wave, which enabled testing centers to manage the surge in demand during the Delta wave.

Despite these adaptations and learnings, respondents suggested several areas where more significant adaptation and learning should have taken place. First, and most critically, private providers felt the government was underprepared because it had not anticipated that the second wave would be so different from the first.

‘PPE [personal protective equipment], isolation practices, hand washing, and masking were the main challenges during the first wave, the second wave was about hospital beds, ventilation, and critical care management… new complications.’ (Private provider 04, Prayagraj)

Given experiences with the Delta variant across the globe, respondents argued that government should have better anticipated these challenges, particularly in terms of the scale of demand for ventilators and oxygen. Second, while the private sector was deeply involved in the response during the Delta wave, respondents from the sector often felt that they had negligible engagement in the policy process, and earlier engagement would have made them better prepared. Instead government waited until it was overwhelmed prior to seeking to empanel more private hospitals in the response. More broadly, access to resources in rural settings remained constrained due to weak primary health care infrastructure.

### Integration

Many private-sector respondents acknowledged the efforts that government had made to respond to the Delta wave, and appreciated the collaborations with government. This was particularly so for diagnostics, where both government and private companies reported strong communication between sectors. Further, the urgency of the situation frequently helped to cut through typical bureaucracy:

‘Our papers weren’t stopped for that. Nothing was kept pending. We were given the approval immediately, ‘Yes, you start it. If you wish to create more such booths then you can do that too.’ Government supported us a lot this way.’ (Private provider 02, Prayagraj)

Like the first wave, the second wave saw strengthened communication networks between public and private sectors particularly at the district-level. Professional associations played a key role in facilitating such communications. In one district for example, the IMA worked with the district administration to plan for preparedness for future waves. However, this was not typical, and as already noted, many private-sector respondents felt that they were brought into discussions too late. Private-sector respondents acknowledged that they were also at fault in this regard:

‘There were many scientific associations. None of the associations, physicians, surgeons, or pediatricians made a presentation to the government that ‘it is not the way you think it is, the wave is going to come soon’. I think the scientific community ignored standard wisdoms.’ (Private provider 04, Prayagraj)

A state-level government stakeholder similarly acknowledged that the response during the second wave was largely reactive because the surge in cases was unexpected and required quick fixes:

‘The reaction of government was more of a knee-jerk reaction… and it led to a situation wherein enough thought was probably not given on how the details of that engagement had to be put in private and public sector. So even though the SOPs [standard operating procedures] and guidelines have been published, there was no hand-holding support for the private sector to ensure that those guidelines are followed and there was no specific monitoring also done for the same. In hindsight we can say that probably we could have worked on those aspects as well, but at that point of time there was not enough time for us to train up all the workforce.’ (Government stakeholder 01, State-level)

Finally, the close collaboration between public and private sectors did not extend to RMPs and other informal health care workers, who were excluded from the response, despite the fact that they are the first resort for most rural, poor households.

## Discussion

Enhanced integration, whether between public and private sectors, or different functional sectors within government, is often viewed as a hallmark of resilience (Hanefeld et al. 2018). In UP, the integration of the private for-profit health sector during the second wave of COVID-19 strengthened the overall resilience of the health system, resulting in coordinated resource management and effective care for those who could access it. However, there were also notable gaps that hindered equitable access and effective service delivery.

The private sector’s role expanded during the second wave. First, rather than only providing surge capacity, private hospitals were proactively integrated through government orders as primary COVID-19 treatment centers. Second, integration was evident in the flow of data and coordination; the private sector was expected to report on COVID-19 case data and oxygen usage, and based on our findings, they largely complied. This allowed the government to allocate critical resources, like oxygen, based on need, and fulfill its stewardship role by referring patients to appropriate health facilities. A third example of integration was the increased coordination with state-level allopathic health providers’ associations, who provided ad hoc input into policy decisions during the peak of the second wave. This inclusion of private-sector voices in the decision-making process helped shape public-health responses in the second wave, highlighting a key adaptation in the government’s approach compared to prior to the second wave. At the same time, the government’s regulatory oversight of the private sector through data-reporting requirements, allocation of critical resources, and pricing caps also prompted and influenced private-sector responses, leading to adjustments to their pricing strategies and fees for COVID-19 care and treatment, for example.

More structured models, such as the National Tuberculosis Elimination Programme (NTEP), demonstrate how engagement and integration of the public and private sectors can be institutionalized. Through NTEP, the government has established a digital case-notification portal called Nikshay, which requires all providers, including public and private hospitals, laboratories, and pharmacies, to register and report tuberculosis cases (Directorate General of Health Services Ministry of Health & Family Welfare Government of India 2024). The platform also links tuberculosis patients to the Nikshay Poshan Yojana, a monthly case-transfer scheme to cover their nutritional needs. This model of collaboration demonstrates how digital platforms can help institutionalize and facilitate public–private partnerships.

Similarly, Suseela and Shannawaz (2023) describe a number of mechanisms to strengthen engagement with the private sector, including establishing formal platforms to coordinate with a wide range of providers, including informal providers, who often serve as the first point of care. Engaging them has been emphasized as a strategy to build trust, strengthen integrated disease surveillance and response, and promote data use for decision-making (Pai 2015, Kelamane et al. 2021).

Consistent with this approach, the World Health Organization recognizes the private sector as an important source of care in LMICs (Grépin 2016), and has emphasized drawing on the private sector’s resources and capacities to support a ‘whole-of-government’ and ‘whole of society’ approach (The World Health Organization 2021). Despite the UP government’s efforts to integrate, at times, the response was siloed or disjointed, which affected equitable access to services, particularly for rural populations.

While larger private hospitals continued to be integral to the response, RMPs remained excluded, despite earlier findings from the first wave showing their primary role in providing care to rural populations (Meghani et al. 2022). Similarly, AYUSH providers, who typically serve lower socioeconomic populations in urban and peri-urban areas, only had limited and ad hoc engagement with district health authorities, further raising equity concerns (Rao et al. 2021, Meghani et al. 2022). Gaps in engagement and coordination between the government and a diverse range of private providers also reflected broader challenges in managing the ‘infodemic,’ which may have reduced the reach and consistency of accurate public health messages (World Health Organization 2025).

A related challenge was the government’s limited capacity to adequately regulate private-sector pricing. Breaches on price caps of COVID-19 services, and reports of private hospitals manipulating claims and charging patients for services not rendered were documented. Confusion over the eligibility of PM-JAY benefits further revealed gaps in the integration of the private sector into the government schemes, which left patients without equitable care. Mistrust between the public and private sectors deepened when families of private health workers who died from COVID-19 were not compensated under PM Gareeb Kalyan Yojana. Together, the nature of these interactions between the public and private sectors suggests that the memorandums of understanding may have been ad hoc, based on one-off directives or agreements. Such approaches likely limited accountability and consistency in implementation, in contrast to the more codified public–private partnership structures established under NTEP, which were built and institutionalized over time.

Similar challenges have been reported in other states in India, where the private sector’s participation in publicly funded health insurance schemes like PM-JAY remains limited due to low or delayed reimbursement rates and difficulties with patient pre-authorization and claims processing, all of which hinder the effectiveness of the scheme (World Health Organization 2023, Marathe et al. 2025). Private hospitals have been accused of profiteering across India (Kumar and Gettleman 2021), with reports of hospitals maintaining high profit-margins despite the emergency setting (Sharma 2022, Dreher 2023). Studies in other states have also documented evidence that patients were overcharged for COVID-19 services, highlighting the limited effectiveness of regulatory measures (Marathe et al. 2023). While many private hospitals, including in UP, argued these charges were justified because of the higher costs of providing COVID-19 services, particularly compensating heath workers at higher rates because of their heightened exposure, the lack of adequate regulatory oversight disproportionately affected poorer populations, exacerbating the inequities in access to essential health services during the pandemic (Williams et al. 2021), which were likely compounded by gender and other social determinants of health (Raghuvanshi et al. 2025).

In contrast to UP’s experience, Kerala’s experience offers a counterpoint. Kerala has a long history of partnering and co-creating services with non-state actors, and the relational trust built over time facilitated a more coordinated and adaptive pandemic response between the public and private sectors (Centre for Public Policy Research (CPPR) 2020). Lessons from tuberculosis care (Khobragade et al. 2022), and from Kerala's broader pandemic response (Prajitha et al. 2021) demonstrate that strong partnerships between the public and private sectors can create synergies that strengthen pandemic preparedness. In turn, these partnerships can also minimize perceptions of, or actual disruptions from new public sector regulations among private- health care providers.

UP has begun taking steps towards strengthening coordination with the private sector, aligned with the broader vision of the National Health Policy 2017 goals to strengthen public and private partnerships (Ministry of Health and Family Welfare Government of India 2017). One example is the Unified Disease Surveillance Platform, a digital system that integrates data on notifiable diseases from public and private providers to support real-time monitoring and surveillance in order to improve coordination and response to health emergencies (Department of Medical Health & Family Welfare Government of Uttar Pradesh and Directorate General of Medical Education & Training Government of Uttar Pradesh 2025).

Looking ahead, establishing public–private partnership coordination units or task forces could further strengthen local stewardship and provide an ongoing platform for collaboration during current and future health emergencies. Building trust and coordination between the public and private sectors is essential and requires an integrated approach that makes health systems more resilient and helps ensure equitable access to care during crises.

Box 1 distills key actions and recommendations, aligned with WHO principles (The World Health Organization 2021), towards building a comprehensive healthcare network that serves all populations, including those in rural and underserved areas.

Box 1. **Summary of recommendations**

To strengthen collaboration between the public and private health sectors and improve preparedness for future health emergencies, the following actions are recommended.

- Expand the role of private providers in both the immediate response and long-term health systems planning.
- Ramp up state capacity to better engage and regulate the private sector through decentralized platforms such as district private–public partnerships units to coordinate, oversee collaboration, and ensure sharing of accurate public-health messages.
- Address price gouging and strengthen accountability through better regulation and enforcement of price caps, and specifically, formalize public-private partnerships with pre-approved contracts, pricing agreements, and data-sharing protocols between government and private providers.
- Clarify and expand insurance coverage to ensure private sector services are covered under schemes like PM-JAY and include transparency reimbursement and claims processes.
- Support ongoing engagement of private-sector providers through peer-learning (Lucas 2012) or communities of practice to foster learning and knowledge sharing (Bennett et al. 2014).
- Promote adaptive learning by documenting and applying lessons from previous health emergencies to strengthen preparedness and inform future responses.
- Invest in maintaining an updated registry of private providers to address information gaps in national health systems (AbouZahr and Boerma 2005).

### Limitations

The primary limitation of our study was that we were only able to interview a limited number of RMPs through an informal providers’ health association. While our team travelled to predominantly rural areas to find RMPs, it was challenging to do so due to their lack of registration with formal authorities. Despite this limitation, our engagement with the respondents over two rounds of interviews facilitated rapport building and illuminated nuances in our analysis, which would not have been possible otherwise. We acknowledge that some media articles may emphasize more prominent events and may not be able to provide sufficient depth or context needed for analysis and interpretation; however, by including both national and local news outlets, we observed consistent reporting across multiple sources, which suggests that the findings reflect broader patterns rather than isolated instances.

## Conclusion

The study illustrates both the successes and limitations of integrating the public and private sectors during the Delta wave of the COVID-19 pandemic. The integration of private hospitals into the public-health response are clear examples of resilience. However, significant gaps in private-sector regulation, and failure to include key providers (RMPs, AYUSH clinics) in ongoing engagement and communication, show where the system failed to fully integrate or adapt. Addressing these gaps requires better integration of the private sector into the public-health response, which can help secure more equitable access to care for all populations.

## Supplementary Material

czag001_Supplementary_Data

## Data Availability

The data underlying this article cannot be shared publicly to protect the privacy of the individuals who participated in the study. The data will be shared on reasonable request to the corresponding author.

## Reflexivity statement

Our team includes collaborators who either reside in or have roots in India, and have extensive research and practice experience in the state where the research is being conducted.
