# Supplementary material for: Health systems resilience and private for-profit sector engagement: lessons from the second COVID-19 wave in Uttar Pradesh, India
Source: Health Policy Plan. 2026 Jan 13;41(4):529–41. doi: 10.1093/heapol/czag001 (PMC13089481; doi:10.1093/heapol/czag001)
Supplement: czag001_Supplementary_Data [file czag001_supplementary_data.docx]

**Supplementary data 1.** Items extracted by category for the news media analysis

| Category | Items extracted |
| --- | --- |
| Article details | - Type of news article [e.g, News, letter to the editor, editorial, op-ed] - Title - Author name: - Date: - URL: |
| Type of private sector discussed | - large corporate private hospitals - (smaller hospitals and nursing homes; - (single-doctor clinics of allopathic providers - practitioners of AYUSH; - pharmacists/private labs; - rural medical practitioners/informal private providers; - nurses, paramedical and other health sector staff; - unspecific; - all (i.e., at least 2 of the above); - other (to describe in separate column) |
| Policy guidelines/policy initiatives/memos discussed | - Level [national, state, district] - Type of policy guidelines [memo, formal gov’t order, etc.] - Date of policy guidelines - Indicate if policy guideline is new or a revision to the previous one - Description of policy guidelines - [describe how it relates to the private health sector or maybe applicable to both private and public sectors] - Indicate implementation agency/any stakeholders - Indicate state of implementation/implementation challenges |
| Patients’ experiences in accessing care | - Description of who (patient or their family/friends) is involved in accessing care [e.g., gender/age/occupations, poor, rural, disadvantaged]: - Patient’s condition/health outcome - Describe experience accessing care |
| Role of profiteering (Yes/no): | - Type of profiteering in the health system - [e.g., sales of fake drugs, selling old fire extinguishers as oxygen canisters, selling health commodities at exorbitant prices, selling access to hospital beds, stealing unused supplies from hospitals] - Description of profiteering [and discussion of any related policy guidelines] [e.g., online brokered deals, scams on social media websites like Twitter, different stakeholders involved] - Impact of profiteering on patients’ lives and their families - [including financial implications, distress, etc.]: - Other |
| Quotes | - Content of quote (including “name”/description of person quoted) |
